# Registered nurse effect on long length of stay in the heart failure hospitalizations of African Americans

**DOI:** 10.1371/journal.pone.0329602

**Published:** 2025-08-05

**Authors:** Tremaine B. Williams, Pearman Parker, Milan Bimali, Maryam Y. Garza, Alisha Crump, Taiquitha Robins, Emel Seker, Ava Storey, Allison Purvis, Mya Tolbert, Anthony Drake, Taren Massey Swindle, Kevin Wayne Sexton

**Affiliations:** 1 Department of Biomedical Informatics, University of Arkansas for Medical Sciences, Little Rock, Arkansas, United States of America; 2 College of Nursing, University of Arkansas for Medical Sciences, Little Rock, Arkansas, United States of America; 3 Department of Biostatistics, University of Arkansas for Medical Sciences, Little Rock, Arkansas, United States of America; 4 Department of Population Health Sciences, University of Texas Health Science Center at San Antonio, San Antonio, Texas, United States of America; 5 Division of Academic Pathways and Workforce Partnerships, University of Arkansas for Medical Sciences, Little Rock, Arkansas, United States of America; 6 Department of Pediatrics, University of Arkansas for Medical Sciences, Arkansas Children’s Research Institute, Arkansas Children’s Nutrition Center, Little Rock, Arkansas, United States of America; 7 Department of Surgery, Vanderbilt University Medical Center, Nashville, Tennessee, United States of America; 8 Department of Biomedical Informatics, Vanderbilt University Medical Center, Nashville, Tennessee, United States of America; University Medical Centre Ljubljana (UMCL) / Faculty of Medicine, University Ljubljana (FM, UL), SLOVENIA

## Abstract

African Americans experience approximately 2.5 times more heart failure hospitalizations than Caucasians and the complexity of heart failure requires registered nurses to work in collaboration with other types of healthcare professionals. The purpose of this study was to identify care team configurations associated with long lengths of hospital stay in African Americans with heart failure hospitalizations and the related effect of the presence of registered nurses on their length of hospital stay. This study analyzed electronic health record data on the heart failure hospitalizations of 2,274 African American patients. Binomial logistic regression identified the association between specific care team configurations and length of stay among subgroups of African American patients. Of the significant team configurations, a Kruskal-Wallis H test and linear regression further assessed the team composition and the specific change in days associated with a one-unit change in the number of registered nurses on a patient’s care team. Six team configurations were associated with a long length of stay among all African Americans regardless of age, sex, rurality, heart failure severity, and overall health severity. The configurations only differed significantly in the proportion of registered nurses with respect to other care team roles. An increase in one additional registered nurse on a care delivery team was associated with an increase in length of stay of 8.4 hours (i.e., 504 minutes). Identifying the full range of social and technical care delivery tasks performed by RNs, and controlling for their effect on length of stay, may be a key strategy for reducing length of stay and explaining why these six configurations and RNs are associated with long LOS. The identification of these models can be used to support decision-making that optimizes the availability of patient access to high-quality care (e.g., clinical staffing and supplies).

## Introduction

Heart disease is the leading cause of death in the United States, affecting 6.2 million people [1]. African Americans have a disproportionately higher burden of modifiable risk factors of heart disease (hypertension and obesity), a higher likelihood of developing heart failure from non-ischemic causes (e.g., genetics), and less access to high-quality heart failure care [2–4]. African American men and women experience approximately 2.5 times more heart failure hospitalization rates than Caucasian men and women [5]. When hospitalized, African Americans have an ¼ day longer length of stay (LOS) in the hospital than non- African American patients which is a consistent measure of healthcare quality [6,7]. Clinically, the goal is to optimize LOS by reducing a patient’s LOS to the absolute minimal amount of time necessary for high-quality care delivery while achieving stability in the patients’ health [6,7]. Otherwise, patients face risks of iatrogenic complications and hospital-acquired infections [6,7]. Reduced LOS additionally contributes to decreased costs and utilization of hospital staff, allowing hospital staff to provide care for other patients with significant clinical needs [6].

Innovatively, system-level strategies such as the implementation of interprofessional care teams (consisting of nurses, physicians, physical therapists, etc.) have been used to optimize LOS [1,8–11]. Interprofessional care teams have been associated with improved outcomes among racial groups, including a 30% decrease in hospitalization odds and 28–31% decrease in 30-day readmission odds over a 7-year period [8–11]. Notably, registered nurses (RNs) have been consistently identified as the most engaged members of interprofessional care teams and have been consistently associated with improved outcomes, including an 88% decreased hospitalization odds in the severest cases of heart failure and a 98% decreased hospitalization odds in multimorbid patients with heart failure [9,12]. While the specific influence of RNs on the LOS of hospitalized African Americans with heart failure is unknown, these improvements in hospitalization odds have been attributed to the scope of work performed by RNs [9–13]. The broad scope of work performed by RNs includes a full range of social and technical tasks (documenting, retrieving, and sharing clinical assessments and triage data within electronic health record systems; conducting procedures and laboratory draws; providing heart failure-related education to support self-management of heart failure; remote monitoring of heart failure; and coordinating care among various providers, specialties, and health systems) [9,13–16]. This scope of work uniquely positions RNs to reduce heart failure hospitalizations through direct intervention by facilitating access to interprofessional team members.

Improved access to interprofessional team members allows for the adjustment of treatment plans, improved heart failure literacy, and arranging transportation to follow-up appointments [17–21]. The presence of RNs on the care team may increase access to high-quality care (i.e., timely access to a clinical workforce that is “capable, qualified, and culturally competent”) and evident in the RN’s association with improved outcomes of hospitalized African Americans with heart failure [22]. However, the complexity of heart failure care requires RNs to work in collaboration with other types of care team roles including, physicians, social workers, and physical therapists [9,13]. Yet, the optimal structure of the care team (i.e., the specific combinations of care team roles that should work with RNs) has not been determined by empirical methods. By identifying the interprofessional team configurations associated with long LOS in African Americans, hospitals can ensure the availability of patient access to high-quality care (e.g., clinical staffing and supplies) and optimize team configurations that decrease LOS. Therefore, the purpose of this study was to identify team configurations associated with a long LOS in the heart failure hospitalizations of African Americans and the related effect of the presence of RNs on their LOS. Additionally, the influence of patient intersectionality (i.e., their sex, rurality, overall health, and heart health) was assessed within team configurations associated with long LOS.

## Methods

### Study design

This retrospective, cross-sectional study analyzed deidentified electronic health record data extracted from the Arkansas Clinical Data Repository which included all hospitalizations from the only academic medical center in the state of Arkansas [23]. The University of Arkansas for Medical Sciences is a 535-bed hospital that includes 431 adult beds, 64 newborn bassinets, and 40 psychiatry beds. This study focused on the hospitalizations of adult African Americans with heart failure ranging from January 1, 2014, to December 31, 2023. A hospitalization was defined as an inpatient admission for a continuous stay. The data were accessed for research purposes on February 27, 2024. The data included all study variables related to each patient’s hospitalization. Patient data included pseudonym identifiers, demographics, clinical diagnoses, medical histories, and comorbidities based on each patient’s International Classification of Disease Codes Version 10. The authors did not have access to information that could have identified individual participants during or after data collection.

**Fig 1:**
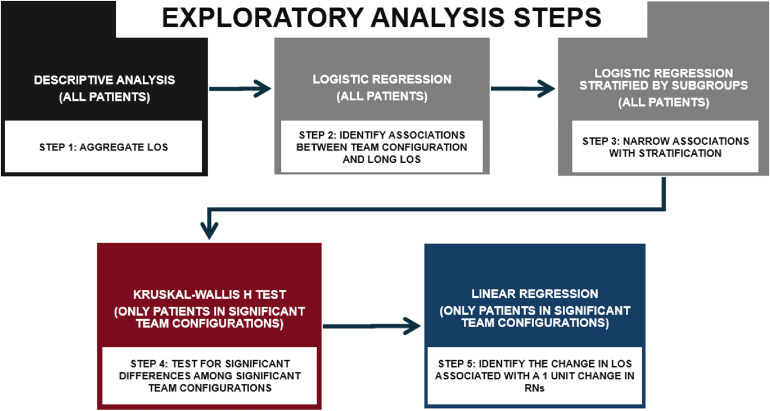
Workflow of exploratory analysis.

Logistic regression identified how the configuration of the care delivery teams of African Americans were associated with long LOS. The logistic regression analysis was further stratified to assess the influence of an African American patient’s sex, rurality, overall health (Van Walraven Elixhauser Comorbidity Score), and specific heart health and function (ejection fraction). This additional analysis was performed because prior studies have shown that patents above age 65, patients who are female, patients with poor overall health, and patients with severe heart failure have increased odds for long LOS [1,3–9,23,25]. The Kruskal-Wallis H test further assessed significant differences in the density of each role within the care team configurations. Subsequently, linear regression identified the change in LOS associated with a one-unit change in RNs (i.e., adding or removing one RN) among statistically significant team configurations. Fig 1 provides a workflow and sequence of the exploratory analysis within the study design. The structure and components of the study design was guided by findings and needs identified in prior studies [9,23].

### Participant selection and sampling

**Fig 2:**
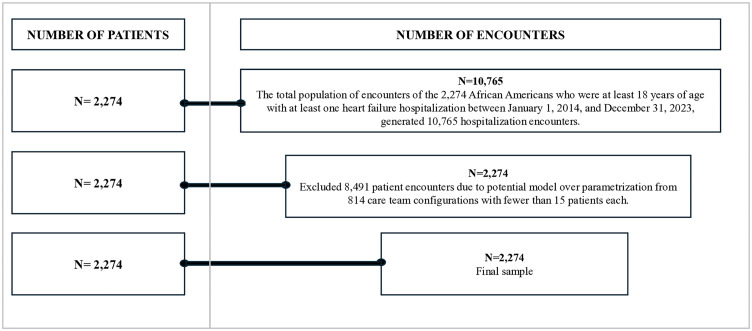
Sample size and participant selection.

The study data only included the hospitalizations of African Americans who had a heart failure hospitalization between January 1, 2014, and December 31, 2023. This 10-year period was selected because of data availability. The analysis included all individuals who were African American and 18 years of age and older because they are disproportionately impacted by higher heart failure hospitalization rates and longer lengths of stay than other patients [2–5]. All patients in the dataset had a diagnosis of congestive heart failure, as characterized by left ventricular ejection fraction via transthoracic echocardiogram. Only hospitalizations with at least one or more of the following care roles were included in the analysis because they were previously established as being associated with hospitalization rates and LOS: physician, nurse practitioner, registered nurse, patient care technician, physical therapist, occupational therapist, respiratory therapist, pharmacists, registered dietician, care manager, and social worker [9,12]. Care team configurations with fewer than 15 patients were excluded from the analysis because of the potential for model over parametrization, which is consistent with prior research [9]. All data that met sampling criteria was analyzed. Fig 2 provides a diagram of how the inclusion and exclusion criteria impacted the sample size.

### Overall health severity

The Van Walraven Elixhauser Comorbidity Score was also provided for each hospitalization. The Van Walraven Elixhauser Comorbidity Score is a single weighted, numeric score based on each patient’s specific combination of comorbidities and their related mortality risk, an overall measure of health severity during the hospitalization [23–27]. The Van Walraven Elixhauser Comorbidity Score is a scoring system ranges from negative twelve to positive eighty-nine, with the lowest quartile (−12–7) representing patients with the best overall health, and patients with scores ranging from eight to eighty-nine indicating patients with poor overall health [23–27]. Higher Van Walraven Elixhauser Comorbidity Score are interpreted as having a higher likelihood of increased LOS and in-hospital mortality [23–27]. The Van Walraven Elixhauser Comorbidity Score has been previously validated through prior international studies in patients with chronic conditions including congestive heart failure [24–27]. It has also been validated through prior work of the authors which specifically focused on patients with congestive heart failure of all races in Arkansas including those who were African American [23]. These studies have concluded that the Van Walraven Elixhauser Comorbidity Score is a valid and reliable tool for isolating high-risk patients and measuring overall health severity related to the odds of in-hospital mortality [23–27].

### Heart failure severity

The dataset also included the ejection fraction rate of each patient as a specific indicator of heart health (the percentage of blood pumped through the left ventricle of the heart) [9,23]. Based on how the patients clinically presented during their hospitalization, patients with less than a 50% ejection fraction reflected cases of heart failure that were the most severe, compared to those with an ejection fraction of 50% or greater [9–11,23].

### Care team configuration

An edge list was also provided within the dataset. The edge list linked each patient with each member of the care team who provided their care. The data related to care team members included pseudonyms and care roles by licensure (e.g., physicians, registered nurses, social workers). Unique combinations of the care team roles that provided care were defined as care team configurations.

### Care team network density

Network density (a method of social network analysis) was used to map and quantify the relationships between patients and their care team members within each the hospitalization [23,28,29]. Network density represented the number of connections each patient had with each care team member divided by the total number of possible connections the patients could have had with all care team members in the network on the specific day of their hospitalization [29]. Network density values (i.e., ranging from 0–1) represented the percentage of available care team members on each hospitalization date who provided care to each patient (i.e., the percentage of available hospital staff used by each patient) [29]. Network density values were calculated for the overall interprofessional mix of all care roles on a care team (e.g., physicians, nurses, and social workers) who provided care for each patient [23]. Network density values were also calculated by care role (e.g., RN network density) to indicate the percentage of available hospital staff in each care role (e.g., RNs) that was used by each patient.

### Statistical analyses

The thresholds presented in Table 1, as well as the dichotomization of age, ejection fraction, Van Walraven Elixhauser Comorbidity Score, and LOS, was consistent with previous studies that applied similar methods, the clinical evaluation of heart failure, and the statistical assumption of the logistic regression analysis (*discussed below*) [1,9,23,30–34]. Table 1 provides a list of dichotomized variables.

**Table 1:** Dichotomous variables.

Original Variable	Original Value	Dichotomized Variable Name	Dichotomized Variable Definition and Thresholds	Dichotomized Value
**Age**	≥ 18	High Age	Above the median age of 64.	1
	≥ 18	Low Age	At or below the median age of 64.	0
**Length of Stay**	>0 days	Long length of stay	A length of stay above the median of 2.11 days.	1
	>0 days	Short length of stay	A length of stay below the median of 2.11 days.	0
**Ejection Fraction** (Heart Failure Severity)	15 to 100%	Relatively poor heart health	≤ 49% of the total blood in the heart that is pumped out.	1
	15 to 100%	Relatively stable heart health	≥ 50% of the total blood in the heart that is pumped out.	1
**Van Walraven Elixhauser Comorbidity Score** (Overall Health Severity)	−19–89	Low mortality risk (Relatively stable health)	The patient had a score that ranged from −19–7.	1
	−19–89	High mortality risk (Relatively poor health)	The patient had a score that ranged from 8 to 89.	1
**Rurality**	1–9	Not rural	The patient resided did not reside in a rural area.	0
	10	Rural	The patient resided in a rural area.	1

Upon receiving the deidentified dataset, Open Refine 2.0 was used to confirm that there was no missingness in the dataset. The data was analyzed using SPSS Version 29. Means, standard deviations, medians, interquartile range, minimum values, and maximum values were calculated to describe LOS by patient characteristic and care team configuration. Logistic regression was used to evaluate how the configuration of the care delivery team of patients was associated with long LOS. Logistic regression was used because the dataset violated the traditional assumptions of parametric tests including normality in the distribution of length of stay, which is consistent with the methodology of similar studies [9,23]. As standard interpretation of logistic regression, the presence of statistically significant odds ratios (p < 0.05, 95% CI) identified team configurations that were associated with long LOS [23]. The regression model tested each team configuration against a reference group which included all patients except for the patients that received the specific team configuration that was being tested (forming two groups: patients who were cared for by the configuration versus patients who were not cared for by the configuration) [9,10,23]. We further tested the extent to which the association between LOS and team configuration held against all patient characteristics (i.e., when stratified by five subgroups of patient characteristics): sex (males and females), age (< 65 and ≥ 65), rurality (resided in rural and non-rural areas), heart failure severity (≤49% and ≥50%), and overall health severity (a Van Walraven Elixhauser Comorbidity Score of ≤ 7 and a Van Walraven Elixhauser Comorbidity Score of > 7).

Two previously established and widely accepted thresholds were assessed to ensure the efficacy of each logistic regression model: the Chi-square omnibus test of model coefficients (assessing overall good model fit) and the area under the curve [6,10,11,23]. A statistically significant Chi-square omnibus test of model coefficients (p-value < 0.05) was used to determine if a regression model with team configuration as a predictor was able to predict the categories of the dependent variable (e.g., long LOS *vs.* short LOS) better than a regression model without the team configurations as included as a predictor. The discrimination ability of the model was evaluated using the area under the curve. Models with an area under the curve value greater than or equal to 0.70 had acceptable discrimination [11]. Subsequently, network density values assessed the extent to which LOS differed significantly among care teams with a larger proportion of a specific care role. Of the team configurations that were significantly associated with LOS after being stratified by age, sex, rurality, heart failure severity, and overall health severity, a Kruskal-Wallis H test assessed significant differences in the LOS, network density, and the number of care team members by role (e.g., the number of RNs on a team within a team configuration). Finally, a linear regression was used to identify the change in LOS associated with a one-unit change in RNs (i.e., adding or removing one RN) within the team configurations which were significantly associated with LOS after being stratified by patient characteristics.

### Ethics statement

The study protocol (#276211) was reviewed and approved by the Ethics Committee of the University of Arkansas for Medical Sciences’ Institutional Review Board. This study was conducted in accordance with all applicable government regulations and institutional research policies and procedures at the University of Arkansas for Medical Sciences. The study team only had access to deidentified data on patients and clinicians. This study presented only minimal risk for loss of confidentiality and a waiver of documentation of the informed consent process was granted by the Ethics Committee, which is the standard for retrospective analyses involving deidentified data. While the Ethics Committee provided a waiver of the informed consent process for this retrospective analysis, the study team recognized the ongoing ethical dialogue regarding patient consent in secondary data use in the era of artificial intelligence. However, artificial intelligence was not used in this study. The Ethics Committee and the study team believed consenting the 2,274 African American patients and their 11,448 care team members would have substantially increased the study’s principal risk for loss of loss of confidentiality due to the need to give the study team access to identifying patient and clinician information solely for the consent process.

## Results

A total of 2,274 African Americans (and their hospitalization) met the inclusion criteria. Table 2 provides demographic information on all African American patients.

**Table 2:** LOS stratified by patient characteristic (N = 2,274).

Demographic Variables	Number of Patients (%)	LOS Mean (SD)	LOS Median (IQR)	LOS MIN – MAX
All Patients	2,274 (100%)	3.28 (4.45)	2.11 (3.98)	0.01-64.87
*By Age*				
-Below 65	1,202 (53%)	3.20 (4.73)	1.99 (3.86)	0.01-64.87
-65 and older	1,072 (47%)	3.37 (4.12)	2.21 (4.30)	0.01-44.41
*By Sex*				
-Male	1,035 (46%)	3.12 (3.94)	2.07 (3.86)	0.01-50
-Female	1,239 (54%)	3.41 (4.84)	2.14 (4.18)	0.01-64.87
*By Rurality*				
-Resided in a rural area	611 (27%)	3.48 (4.09)	2.22 (4.20)	0.01-28.06
-Resided in an urban area	1,663 (73%)	3.21 (4.58)	2.07 (3.93)	0.01-64.87
*By Heart Failure Severity (ejection fraction)*				
-Reduced and mildly reduced (≤ 50%)	148 (6.5%)	5.77 (5.85)	3.86 (3.88)	0.06-28.09
-Preserved (≥ 50%)	130 (6%)	5.83 (7.74)	3.83 (3.90)	0.31-64.87
*By Overall Health Severity (Van Walraven Elixhauser Comorbidity Score)*				
-Low mortality risk (−19–7)	418 (18%)	2.27 (3.21)	1.25 (4.35)	0.01-26.89
-Medium mortality risk (8–34)	1,753 (77%)	3.37 (4.43)	2.20 (4.14)	0.01-64.87
-High mortality risk (35–61)	103 (5%)	5.93 (7.14)	3.44 (3.90)	0.08-33.06
-Very high mortality risk (62–89)	0 (0%)	–	–	–

Values of less than 1 (ex. 0.01) represented a percentage of a 24-hour day.

Abbreviations: LOS, Length of Stay; IQR, Interquartile Range; Max, Maximum Value; MIN, Minimum Value; SD, Standard Deviation.

The 2,274 African American patients were provided care by 11,448 care team members (Table 3). The 11,448 care team members interacted with the 2,274 patients 31,333 times during their hospitalizations. The RNs were the largest care role (n = 7,429 RNs) and the most engaged care role (20,408 interactions) found within the hospitalizations. Patients with a registered dietician on their care team had the highest mean and median LOS (7.88 and 6.21 days, respectively). Patients with a respiratory therapist had the largest network density (0.78).

**Table 3:** LOS stratified by specific care team roles found within the hospitalizations.

Care Team Role Variable	Number of Care Teams (%)	Number of Role	Patient Interactions	Network Density Mean (SD)	LOS Mean (SD)	LOS Median	LOS Min. -Max.
Physician	2064 (90%)	2,746	4,923	0.62 (0.36)	3.34 (4.48)	2.15	0.01-64.86
Nurse Practitioner	500 (21%)	316	845	0.69 (0.33)	5.11 (6.78)	3.03	0-64.86
Registered Nurse	2081 (91%)	7,429	20,408	0.63 (0.35)	3.56 (4.55)	2.42	0.01-64.86
Patient Care Technician	432 (18%)	316	1,113	0.61 (0.36)	6.33 (6.87)	4.14	0.27-64.86
Pharmacist	284 (12%)	18	316	0.73 (0.28)	7.82 (7.15)	5.70	0.55-64.86
Occupational Therapist	397 (17%)	148	822	0.62 (0.36)	7.13 (6.60)	5.34	0.71-64.86
Physical Therapist	579 (25%)	227	1,265	0.72 (0.29)	6.86 (6.43)	5.18	0.53-64.86
Registered Dietician	328 (14%)	73	426	0.74 (0.27)	7.88 (6.94)	6.21	0.92-64.86
Respiratory Therapist	385 (16%)	125	733	0.78 (0.26)	7.48 (7.43)	5.32	0.83-64.86
Care Manager	150 (6%)	16	157	0.72 (0.30)	7.43 (8.18)	5.01	0.67-64.86
Social Worker	271 (11%)	34	325	0.70 (0.29)	6.46 (6.81)	4.30	0.30-60.57

Values of less than 1 (ex. 0.01) represented a percentage of a 24-hour day.

Abbreviations: LOS, Length of Stay; IQR, Interquartile Range; MAX, Maximum Value; MIN, Minimum Value; SD, Standard Deviation.

There were sixteen distinct care team configurations found within the 2,274 hospitalizations (Table 4) that met the study criteria (Fig 2). The team configuration with the largest number of patients was the combination of physician and RN (n = 660 patients; 29% of the sample). The team configuration with the largest mean (5.31 days) and median (4.85 days) LOS was the combination of physician, RN, occupational therapist, physical therapist, and registered dietician. The largest mean network density was found within patients who had a “P+MD+RN+PCT+RT” team configuration (0.84). The smallest mean network density was found within patients who had a “P+NP+RN” team configuration (0.44), which also had the smallest mean and median LOS (0.73 and 0.13 days).

**Table 4:** Network density and LOS stratified by care team configuration.

Care Team Configuration	Patients (%)	Network Density Mean (SD)	LOS Mean (SD)	LOS Median	LOS Min. - Max.
P + NP + RN	56 (2%)	0.44 (0.41)	0.73 (1.91)	0.13	0.01-13.70
P + MD + NP + RN	129 (6%)	0.61 (0.35)	1.83 (1.62)	1.22	0.20-12.07
P + MD + NP + RN + RT	18 (<1%)	0.81 (0.28)	3.78 (2.60)	3.28	0.83-12.05
P + MD + RN	660 (29%)	0.52 (0.38)	0.99 (1.35)	0.26	0.01-8.58
P + MD + RN + CM	16 (<1%)	0.55 (0.34)	3.39 (1.93)	2.99	0.67-8.42
P + MD + RN + OT + PT	56 (2%)	0.59 (0.32)	3.50 (2.48)	2.66	0.82-13.37
P + MD + RN + OT + PT + RD	19 (1%)	0.74 (0.26)	5.31 (2.10)	4.85	2.72-10.73
P + MD + RN + PCT	86 (4%)	0.65 (0.33)	2.75 (1.59)	2.63	0.27-7.72
P + MD + RN + PCT + OT + PT	18 (<1%)	0.67 (0.31)	3.37 (1.87)	3.20	1.10-9.58
P + MD + RN + PCT + RD	17 (<1%)	0.66 (0.30)	4.91 (2.69)	4.23	1.85-13.53
P + MD + RN + PCT + RT	21 (<1%)	0.84 (0.25)	3.43 (1.56)	3.32	0.89-6.05
P + MD + RN+PHARMD	18 (<1%)	0.65 (0.33)	2.96 (1.41)	2.64	0.55-5.27
P + MD + RN + PT	35 (2%)	0.68 (0.31)	3.49 (2.32)	3.02	0.93-12.18
P + MD + RN + RD	30 (1%)	0.72 (0.31)	3.77 (2.06)	3.22	0.92-10.08
P + MD + RN + RT	47 (2%)	0.73 (0.32)	3.31 (2.35)	2.99	1.04-13.21
P + MD + RN + SW	33 (1%)	0.60 (0.33)	2.45 (1.28)	2.42	0.51-5.81

Values of less than 1 (ex. 0.01) represent a percent of a day.

Abbreviations: CM, Care Manager; IQR, Interquartile Range; LOS, Length of stay; Max, Maximum; MD = Physician; MIN, Minimum; NP, Nurse Practitioner; OT, Occupational Therapist; P, Patient; PHARMD, Pharmacist; PT, Physical Therapist; PTC, Patient Care Technician; RD, Registered Dietician; RN, Registered Nurse; RT, Respiratory Therapist; SD, Standard Deviation; SW, Social Worker.

**Fig 3:**
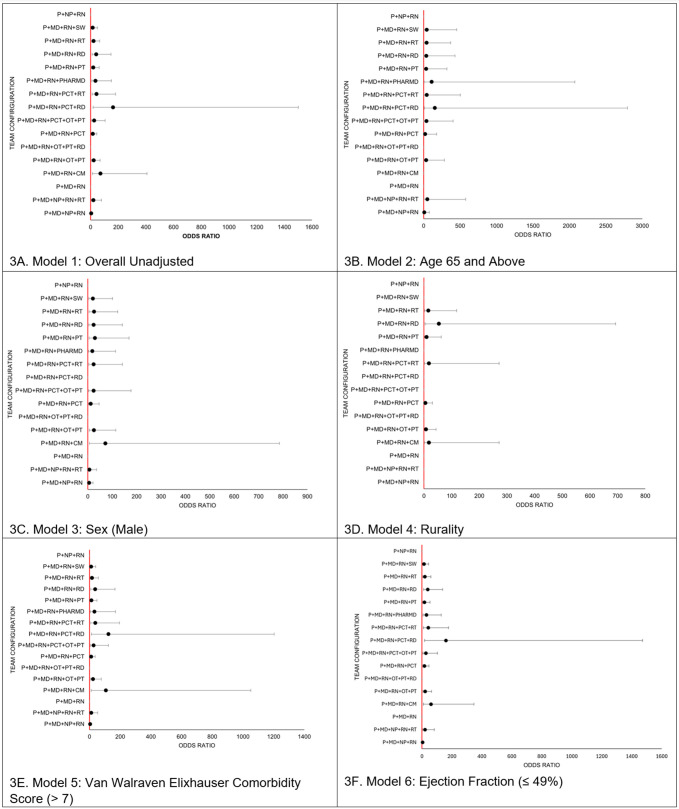
Forest plots of odds ratios of the associations between long LOS and configuration stratified by patient characteristic. (3A-F) Abbreviations: CM, Care Manager; IQR, Interquartile Range; LOS, Length of stay; Max, Maximum; MD = Physician; MIN, Minimum; NP, Nurse Practitioner; OT, Occupational Therapist; P, Patient; PHARMD, Pharmacist; PT, Physical Therapist; PTC, Patient Care Technician; RD, Registered Dietician; RN, Registered Nurse; RT, Respiratory Therapist; SD, Standard Deviation; SW, Social Worker. Odds ratios with a p-value ≥ 0.05 were not included in the plots because they were not statistically significant.

Fig 3 provides all statistically significant odd ratios for African American patients having a long LOS from the logistic regression, and when stratified (by age, sex, rurality, Van Walraven Elixhauser Comorbidity Score, and ejection fraction) to illustrate the team configuration models associated with long LOS. Fig 3A (Model 1) presents the association between the team configuration and long LOS for all African Americans. Fig 3B (Model 2) presents the association between the team configuration and long LOS for African Americans who were age 65 and above compared to African Americans who were below age 65. Fig 3C (Model 3) presents the association between the team configuration and long LOS for African Americans who were male compared to African American females. Fig 3D (Model 4) presents the association between the team configuration and long LOS for African Americans who resided in a rural area compared to African Americans who did not reside in a rural area. Fig 3E (Model 5) presents the association between the team configuration and long LOS for African Americans with relatively poor overall health (a Van Walraven Elixhauser Comorbidity Score that ranged from 8 to 89; high mortality risk) compared to African Americans with relatively stable overall health (a Van Walraven Elixhauser Comorbidity Score that ranged from −19–7; low mortality risk). Fig 3F (Model 6) presents the association between the team configuration and long LOS for African Americans with relatively poor heart health (ejection fraction; ≤ 49% of the total blood in the heart is pumped out) compared to African Americans with relatively stable heart health (ejection fraction; ≥ 50% of the total blood in the heart is pumped out). The Chi-square omnibus tests of each of the six models yielded a p-value of 0.001, indicating good model fit. All models had an area under the curve value of greater than 0.70, indicating an acceptable level of discrimination.

There were three team configurations that were not significantly associated with long LOS in the unadjusted and stratified regression models (“P+NP+RN”, “P+MD+RN”, and “P+MD+RN+OT+PT+RD”). There were also six team configurations that did not lose significance when stratified for age, sex, rurality, Van Walraven Elixhauser Comorbidity Score, and ejection fraction (“P+MD+RN+OT+PT”, “P+MD+RN+PCT”, “P+MD+RN+PCT+RT”, “P+MD+RN+PT”, “P+MD+RN+RD”, and “P+MD+RN+RT”). Notably, all team configurations were associated with increased odds of having a long LOS, including the six team configurations that did not lose significance when stratified for age, sex, rurality, Van Walraven Elixhauser Comorbidity Score, and ejection fraction. Subsequent models did not include covariates from previous models because the models lost significance due to the shrinking sample size. There were no team configurations that were associated with a patient having decreased odds of having a long LOS (a short LOS). Physicians and RNs were the only common care roles found in all six team configurations that held significance after being stratified. The results of these six team configurations found a statistically significant association in all subgroups of patients (males and females; below age 65 and above age 65; resided in a rural area and resided in an urban area; least severe heart failure cases and most severe heart failure cases; and patients with poor overall health and patients with good overall health). All unadjusted and stratified regression models had a statistically significant chi-squared omnibus test of model coefficients (p-value < 0.05), indicating that team configurations were a better fit of long LOS than the null regression models without the team configurations included as predictors.

**Fig 4:**
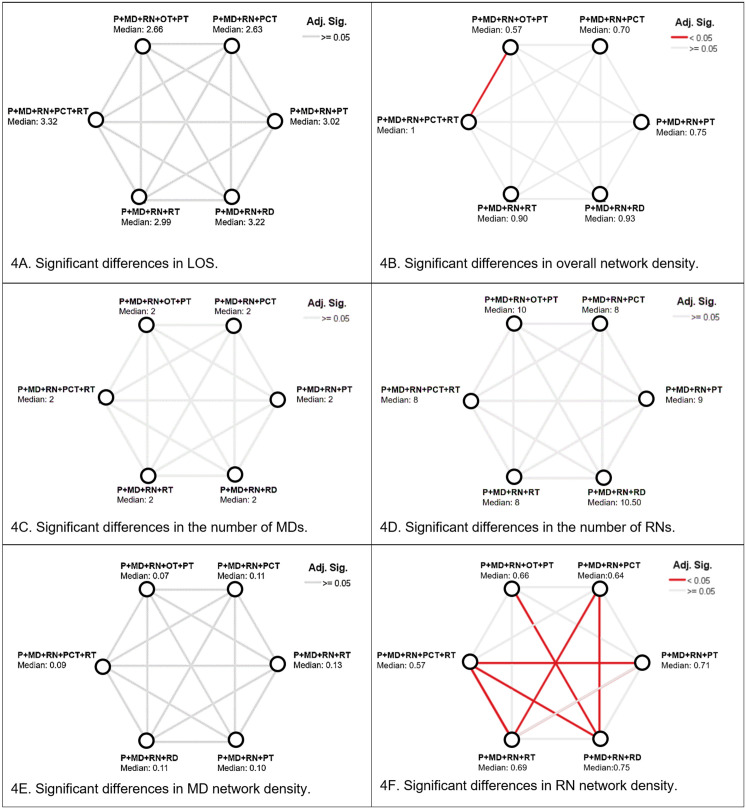
Kruskal-Wallis H test of differences in the six team configurations.

Of the six team configurations that were associated with a long LOS, physicians and RNs were the only common care roles found in all teams. Therefore, we additionally assessed the six team configurations for significant differences in the number of physicians and RNs on each team within each configuration. The Kruskal-Wallis H test determined that there were no significant differences in: 1) the number of RNs among the teams (i.e.,., ranging from 8−11 nurses), 2) the median LOS of patients (2.63–3.32 days), 3) the number of physicians among the teams (2 physicians), nor 4) the physician-specific network density (0.07–0.13). However, there was a significant difference in the overall network density of two team configurations (“P+MD+RN+OT+PT” and “P+MD+RN+PCT+RT”, with 0.57 and 1, respectively). This indicated that patients within the “P+MD+RN+PCT+RT” configuration was provided care for by all the care team members of each care role who were available during the hospitalization, compared to patients in the “P+MD+RN+OT+PT” configuration who were provided care from 57% of the care team members of each care role who were available during the hospitalization. Furthermore, there were significant differences among the six team configurations in their RN-specific network density, the percentage of RNs who composed the teams varied in size with respect to the overall interprofessional mix of other care roles. Visual inspections of boxplots determined that the distributions were approximately similar for all significant configurations (Fig 4).

Within the six team configurations that were associated with long LOS, a linear regression identified the effect of adding one additional RN to a care team on a patient’s LOS. Visual inspection of the scatterplot indicated a linear relationship between the number of RNs on each team and LOS in days. There was independence of residuals, which was assessed by a Durbin-Watson statistic of 1.84. Homoscedasticity was confirmed by visual inspection of a plot of standardized residuals versus standardized predicted values, and visual inspection of a normal probability plot indicated that residuals were normally distributed. The number of RNs on a heart failure patient’s care team accounted for 82.4% of the variation in long LOS with an adjusted R^2^ = 82.3%. The number of RNs on a patient’s care team statistically significantly predicted long LOS (β = 0.35, p < 0.05). An increase of one additional RN on a care delivery team was associated with a 0.35 (95% CI, 0.33 to 0.37) increase in LOS (i.e., 8.4 hours or 504 minutes) among teams associated with a long LOS. Inversely, a decrease in one RN on a care delivery team was associated with a decrease in LOS of 8.4 hours (i.e., 504 minutes). Additionally, the effect of adding and removing an additional physician was also assessed because physicians and nurses were the only roles present in all team configurations. However, there was no statistically significant effect (p > 0.05).

## Discussion

### Summary of findings

Sixteen care team configurations were identified, of which all were associated with increased odds of having a long LOS in African Americans with heart failure. There were no configurations associated with a short LOS. Of the sixteen configurations, six were associated with a long LOS when stratified for age, sex, rurality, heart failure severity, and overall health severity. Within the six configurations, an increase and decrease in one additional RN on a care delivery team was associated with an increase and decrease in LOS of 8.4 hours (i.e., 504 minutes), respectively.

### Implications for research and practice

The results indicate that the configuration of care delivery teams is associated with LOS.

This association implied that team configuration can be used to identify and track African American patients with heart failure who are at increased odds of having a long LOS and increased odds of in-hospital mortality, as evident by the significant associations with team configurations and Van Walraven Elixhauser Comorbidity Score, respectively. All sixteen of the care team configurations that were significant were associated with increased odds of having a long LOS. There were no configurations identified as being associated with a short LOS. This finding indicated that the sixteen configurations assessed were unlikely to be the optimal structure of heart failure care teams for African Americans due to their lack of association with short LOS. Configurations associated with short LOS would have required additional inquiry within experimental designs to narrow configurations for use in optimizing care. Therefore, additional combinations of care roles should be added to roles assessed within the sixteen configurations to explore their association with short LOS and stratified by indicators (e.g., ejection rate, shortness of breath) and pseudo-indicators (e.g., walking without a walker or wheelchair) of relatively good health at discharge [1,15].

Of the sixteen configurations, only six (“P+MD+RN+OT+PT”, “P+MD+RN+PCT”, “P+MD+RN+PCT+RT”, “P+MD+RN+PT”, “P+MD+RN+RD”, and “P+MD+RN+RT”) were associated with a long LOS in African Americans with heart failure when stratified by age, sex, rurality, heart failure severity, and overall health severity. Notably, RNs were the largest care role, and the most engaged care role found within the hospitalizations. Within the context of prior findings, this finding indicates that RNs are positioned to facilitate opportunities for increased inpatient intervention (i.e., heart failure-related education, medication adjustment, laboratory draws, engaging the cardiologists) if used as a new technique for identifying high-need patients in real-time [9,15,35,36]. However, avenues for realizing the real-time identification and management of care delivery team risk scores are needed.

A potential avenue for quantifying the effect of RNs and the teams they work within is social network analysis, as piloted here (i.e., overall network density and RN-specific network density). However, understanding the presence or absence of a specific care team role does not provide sufficient information for developing evidence-based team configurations that drive high-quality care delivery. Social network analysis metrics, including centrality, reciprocity, and transitivity, assess the importance of a team member, linkages, and the likelihood of similar care delivery behaviors among team members [37]. These metrics are critical to understanding how care delivery teams interact and how those interactions influence the outcomes of African American patients with heart failure while the outcomes are being influenced. These metrics can be fueled by metadata that is generally collected in electronic health record systems within the day-to-day workflow of care delivery, data such as who is on a patient’s team. Yet, a range of care delivery factors has historically influenced the LOS and mortality of African Americans, including nursing surveillance, expertise, patient workload, work environment, the quality of interactions with other team members, and clinical decision support. Future research in this domain should focus on providing a deeper understanding of the care delivery team data needed for high-quality nursing care, nurse-related information flow and sharing among care teams, and electronic health record-based communication networks of care delivery teams, of which social network analysis will provide the infrastructure needed to advance [38]. Employing this level of additional investigation will provide specificity on the universal definition of healthcare access, which is limited to a “workforce: [that are] capable, qualified, [and] culturally competent providers” [22]. These findings indicate a need to evolve the definition of healthcare access to include those care delivery team roles that are associated with high-quality care delivery in specific diseases and conditions. Models of care delivery that reduce LOS and mortality in African Americans can be developed to address the disproportionate impact of heart failure on African Americans.

Although the number of physicians, RNs, and the LOS were similar across the six team configurations that were consistently associated with long LOS, the teams differed in the percentage of RNs relative to other roles like physicians or physical therapists. Specifically, these differences were noticeable in how dense RNs were in the overall team’s network of interactions with patients. Some teams had a higher concentration of RNs relative to other roles (i.e., RN-specific network density), which changed how integrated RNs were within interprofessional teams. Notably, more complex patients may have required higher concentrations of RNs relative to other roles. Higher concentrations of RNs relative to other roles could have also occurred because of time. Our approach to additionally calculating network density by care role was innovative and could become a useful and an expanded component of operationalizing social network analysis, as there are no known prior studies that have sought to assess the influence of specific care roles with respect to the overall interprofessional mix of other care team roles to improve the design of care teams [39].

Among teams associated with a long LOS, an increase of one RN on a care delivery team was associated with an increase in LOS of 8.4 hours (i.e., 504 minutes). Inversely, a decrease in one RN on a care delivery team was associated with a decrease in LOS of 8.4 hours (i.e., 504 minutes). Generally, this relationship is explained as the more time a patient spends in the hospital, the more registered nurses would be on their teams. However, our results indicated that the number of RNs on a heart failure patient’s care team explained only 82.4% of the variation in long LOS. This indicates there was an estimated 17.6% of variation that could be due to other explanations.

While this finding advances the understanding of the influence of RNs on the LOS of African Americans with heart failure, neither the current finding nor prior studies provide sufficient information for making evidenced-based decisions about adding or removing a specific number of RNs from a patient’s team. Prior studies have been limited to surveys and retrospective approaches [40]. The interpretation of the association between RN engagement in care and LOS has been largely cross-sectional and limited by an inability to establish that association and/or potentially presume cause (i.e., RNs and their actions related to providing care) precedes the effect (long LOS). The findings indicate a strong potential for prospectively designing care teams to provide high-quality care with the minimum number of RNs needed to optimize LOS, a potential solution for local resource constraints and more global challenges such as the national nursing shortage [41,42]. For example, acute care settings could ensure the specific number of RNs on the care teams of African Americans and the evidenced-based frequency of tasks performed by RNs such as interval clinical assessment, continuing heart failure education, and interval medication adjustment assessment to reduce LOS. However, experimental designs must be employed to control for covariates and confounders when ascertaining the extent to which RNs, team configurations, and their tasks caused short and long LOS. The application of these designs will provide sufficient justification for prospectively designing teams. For example, patients would be provided care for by teams that are not only associated with positive outcomes but also caused them.

## Limitations

The findings of this study were limited by several components of the study design and its data. Principally, the data was generated from only one hospital, Arkansas’ only academic health center, and may not reflect the nature of care delivery in all healthcare systems in the United States. Therefore, the findings may not be generalizable to all care teams and African Americans with heart failure hospitalization in the United States. However, the findings may be generalizable to health systems in states with similar characteristics (e.g., only one academic health center in the state, hospitals caring for African Americans with heart failure hospitalizations in a predominantly rural state) and those health systems whose representation mirrors the characteristics sample of African Americans in this study.

All possible combinations of team configurations were not evaluated due to the types of care team configurations and the number of African Americans that were found within in them, which was limited to the configurations that were formed organically during the standard day-to-day delivery of care. Additionally, African American patients with the highest possible mortality risk (i.e., Van Walraven Elixhauser Comorbidity Score of 62–89) were not evaluated because they were not present in the data. In addition, the study was limited by the sole use of ejection fraction to estimate the severity of a patient’s heart failure. While ejection fraction is the principle physiological indicator of heart failure, additional clinical evaluation of a patient’s heart failure severity (shortness of breath, frailty, exercise tolerance, vital signs, and acuity) would have strengthened the measurement of heart failure severity in this study. Furthermore, the retrospective study design did not support recommendations to add or remove a specific number of RNs or other care team roles to or from care delivery teams to increase or decrease LOS because it did not employ an experimental design with appropriate controls.

## Conclusions

Six interprofessional care team configurations were associated with long LOS in the heart failure hospitalizations of African American with congestive heart failure of all ages, sexes, rurality, heart failure severities, and overall health. The identification of these models can be used to support decision-making that optimizes the availability of patient access to high-quality care (e.g., clinical staffing and supplies). Within these six models, a one-unit change in the number of RNs on a care team was associated with a change in LOS of 8.4 hours. Care team configurations only differed significantly in the percentage of RNs relative to other roles and RNs had the largest number of interactions with patients, suggesting that RNs and the care tasks they performed within multidisciplinary care teams may be the most influential to reducing the length of stay of hospitalized African Americans with heart failure relative to other care team roles. Identifying the full range of social and technical care delivery tasks performed by inpatient RNs, and controlling for their effect on length of stay, may be a key strategy for reducing length of stay and explaining why these six specific configurations and RNs are associated with long LOS.
